# A Noted Pioneer Physician

**Published:** 1912-02

**Authors:** 


### EDITORIAL DEPARTMENT

DR. F. E. DANIEL, Editor. DR. R. H. L. BIBB, Associate Editor.

### A NOTED PIONEER PHYSICIAN.

I am indebted to Mr. Walter Durham of Austin, a grandson of Dr. Lincecum, for the photo reproduced on the opposite page, and for data in the life and experiences of this great man, one of the most remarkable men of the nineteenth century. The older members of the medical profession of Texas will remember him, and recall his contributions to our knowledge of the indigenous medicinal plants of the State. His daughter, Mrs. S. L. Doran of Hempstead, still living, has his Herbarium which he collected at times from 1829 to 1873, the date of his death, and which should be in possession of the U. S. Museum. But I dare say the younger men—those of the present generation and the past one—never heard of him. Such a man and his work should not be forgotten.

Dr. Lincecum was born in Georgia in 1793. His father, Hesekiah Lincecum, a captain in Andrew Jackson's war of 1808-1812, raised the first crop of cotton ever planted in Georgia, and Gideon helped, with all the family, to pick off the lint with his fingers. He saw in operation the first cotton gin made by Whitney in 1802. He seems to have been of a roaming disposition, migrating first to South Carolina, thence to Tennessee, thence back to Georgia, and thence to Tuscaloosa, Ala., thence to the Tombigbee river, called then by the Indians and early settlers "Tombecbee," which was, at that time, a part of what is now Mississippi, and settled opposite where Columbus now stands. He cut his way through the untrod forest, made his own road to reach it. He built the first house in Columbus, and having been appointed chief justice of the region now known as Monroe county and Lowndes county, he laid off the town, sold the lots and built schoolhouses in Columbus. That was November, 1818. Dr. Lincecum was then about 27 years old and had a family. The whole of Mississippi was then owned and occupied by the Chahtas, a nation made up of Choctaws, Cherokees and Creek Indians, under three great chiefs, the most noted of which was the Choctaw, Apushamahatta,—statesman, warrior and powerful orator. The life of this chief by Dr. Lincecum, written first in Choctaw and later translated for and published by the Mississippi Historical Association, is one of the most thrillingly interesting pieces of literature it has ever been my fortune to read.

Dr. Lincecum, during his migrations and his residence in Eastern Mississippi, followed any and every occupation by which he could make a living—hunting for food, farming, day laborer for hire, felling trees, riving boards, sawing out plank with a whip-saw, hewing logs, building houses, keeping "store"—catering to the frontier trade, exchanging goods—largely whisky—for the product of the chase, which he sent to Mobile and Philadelphia—making great profit, teaching school, holding court and *practicing medicine.* He had studied a few medical books, and equipping himself with drugs—bought in Mobile—"allopathic drugs," he called them, and he became known far and near as a successful physician. But he was seduced from the regular practice by the occurrence of an epidemic of dysentery, in which he was very unsuccessful, and adopted the "Thompsonian"* treatment, "decoction of lobelia," "Number 6," etc., and with this he was more successful and won great renown and accumulated money. He gives a ludicrous account of how, in disgust, he dumped his drugs in a pile on the side of the road and of the ebulition that followed, creating a feeling of awe in the ignorant. I wish I had room to reproduce his account of the change of practice. He was in high favor with the Indians, in whose midst he lived a long time. They called him "Shappo-Tohobo" ("white hat") and "Hopi Cheto" ("Big Leader").

In 1835 he rode to Texas on "Ned," his 17-year-old horse, on which he practiced medicine in the then wilderness. Six companions accompanied him to Texas, and starting from San Felippe they explored the country west of the Brazos. His companions deserted him and returned to Mississippi, but the indefatigable doctor remained alone and took a good look at the country, and finally settled with his family in Washington county, in 1848, where he resided and practiced medicine to the date of his death, November 28, 1873.

When it is recalled that at that early date (1835) this country was occupied by the warlike nomadic Comanches it is truly surprising that Dr. Lincecum was not killed by them, and we wonder at his daring hardihood in riding alone all over Southern and Southwestern Texas—and even, later—"exploring twenty-eight counties in Northwest Texas." But he was fearless. Think of a

*Thompson's *Guide to Health* was just published, $20 a copy.

lone man riding and leading a pack mule roaming over the wilds of Texas in 1835 gathering specimens of the flora and fauna of the unknown country—observing and making record of everything and making maps, living on deer and bear meat and fowls, sleeping on a blanket and cooking his food. He put his eye on a splendid piece of land in Washington county, and on his return to Texas with his family in 1848 he bought it and called it "Long Point," where he resided to the date of his death, in 1873.

It seems he did not go into the Confederate army, but aided all he could by donations and care of the soldiers and practicing medicine; he did not, however, practice the Thompsonian method, but returned to his first love—"allopathy"—he called it.

I should not fail to mention that during his early days among the Choctaws in East Mississippi he took a course of instruction from the big medicine man of the Choctaws, Eliche Chito Okla-Hunale. They retired to the depths of the primeval forest on the banks of the Tombigbee, where, for twenty-one days, the old Indian made him acquainted with all the plants used in Indian practice (specimens of which he preserved), all of which he wrote down and later read to his preceptor. On parting he paid the "doctor" $21, which he refused to accept, taking but ten, to buy a pair of blankets at the trading post.

In addition to the life of Apushamahatta Dr. Lincecum wrote (in Choctaw, and later in English) "Choctaw Traditions About Their Settlement in Mississippi and the Origin of Their Mounds," published in *Trans-Mississippi Historical Association,* Vol. VIII.

He sent to Charles Darwin—with whom he corresponded—forty-eight specimens of Texas ants. His work on the ant is classic, authoritative, and is extensively quoted by later writers, notably by Rev. J. F. McCook, in "The Agricultural Ants of Texas." He also sent to the *Jardin des Plantes* of Paris 600 specimens of the flora of Texas, and to the Smithsonian Institution various botanical and entomological specimens, and to the New York College of Science a very large collection of Texas butterflies. Dr. Lincecum spent five years (after the war) in Tuxpan, Mexico. There he made elaborate collections of natural history specimens, which he also presented to various scientific institutions.

The medical profession of Texas can not afford to forget this great man. We should be proud of him and cherish his memory and preserve it for future generations of doctors by some suitable monument or tablet, as an example and an incentive to those who shall come after us.

Dr. A. L. Lincecum of El Campo, Texas, Vice President of the Texas State Medical Association, is a grandson of Gideon Lincecum. He is following in the footsteps of his grandsire and has considerable reputation as an entomologist, and lecturer on the fly.

